# Marker- three dimensional measurement versus traditional radiographic measurement in the treatment of tibial fracture using Taylor spatial frame

**DOI:** 10.1186/s12891-022-05112-3

**Published:** 2022-02-16

**Authors:** Qixin Liu, Yanshi Liu, Hong Li, Xuefei Fu, Xingpeng Zhang, Sida Liu, Jinli Zhang, Tao Zhang

**Affiliations:** 1 Graduate College of Tianjin Medical University, Tianjin, China; 2 Department of Trauma and Microreconstructive surgery, the First Affiliated Hospital of Xinjiang Medical University, Urumqi, Xinjiang China; 3 Department of Orthopedics, Zigong Fourth People’s Hospital, Zigong, Sichuan China; 4 Department of Orthopedics, Anhui No.2 Provincial People’s Hospital, Hefei, Anhui China; 5 Department of Orthopedics, Shanghai Pudong New Area People’s Hospital, Shanghai, China; 6 College of Mechanical Engineering, Tianjin University, Tianjin, China; 7 Department of Orthopedics and Trauma, Tianjin Hospital, Tianjin, China

**Keywords:** External fixation, Taylor spatial frame, Three-dimensional reconstruction, Tibial fracture

## Abstract

**Background:**

The Taylor Spatial Frame (TSF) has been widely used for tibial fracture. However, traditional radiographic measurement method is complicated and the reduction accuracy is affected by various factors. The purpose of this study was to propose a new marker- three dimensional (3D) measurement method and determine the differences of reduction outcomes, if any, between marker-3D measurement method and traditional radiographic measurement in the TSF treatment.

**Methods:**

Forty-one patients with tibial fracture treated by TSF in our institution were retrospectively analyzed from January 2016 to June 2019, including 21 patients in the marker-3D measurement group (experimental group) and 20 patients in the traditional radiographic measurement group (control group). In the experimental group, 3D reconstruction with 6 markers installed on the TSF was performed to determine the electronic prescription. In the control group, the anteroposterior (AP) and lateral radiographs were performed for the traditional parameter measurements. The effectiveness was evaluated by the residual displacement deformity (RDD) and residual angle deformity (RAD) in the coronal and sagittal plane, according to the AP and lateral X-rays after reduction.

**Results:**

All patients achieved functional reduction. The residual RDD in AP view was 0.5 (0, 1.72) mm in experimental group and 1.74 (0.43, 3.67) mm in control group. The residual RAD in AP view was 0 (0, 1.25) ° in experimental group and 1.25 (0.62, 1.95) °in control group. As for the lateral view, the RDD was 0 (0, 1.22) mm in experimental group and 2.02 (0, 3.74) mm in control group, the RAD was 0 (0, 0) ° in experimental group and 1.42 (0, 1.93) ° in control group. Significant differences in all above comparisons were observed between the two groups (AP view RDD: *P* = 0.024, RAD: *P* = 0.020; Lateral view RDD: *P* = 0.016, RAD: *P* = 0.004).

**Conclusions:**

The present study introduced a marker-3D measurement method to complement the current TSF treatment. This method avoids the manual measurement error and improves the accuracy of fracture reduction, providing potential advantages of bone healing and function rehabilitation.

## Background

Delayed bone union and nonunion of open tibial fracture was commonly observed in clinical practice [1, 2]. External fixation plays an important role in the treatment of these problems, providing beneficial microenvironment for fracture healing and including advantages of the management of bone nonunion, osteomyelitis and other diseases [3–7]. However, the traditional Ilizarov system requires a steep learning process [8]. The Taylor Spatial Frame (TSF) was derived from the Stewart platform and Ilizarov external fixator, consisting of two complete or incomplete rings connected by six telescopic struts. The spatial deformities can be simultaneously corrected by the TSF without the alternation of frame configurations [9], and the TSF has been widely used in orthopedic surgeries.

However, parameters of TSF system need to be manually measured on radiographs resulting in subjective errors during the traditional radiographic measurement [10, 11]. In addition, the axial information cannot be obtained from the two dimensional X-rays and it is usually estimated by physical examinations [12]. The aforementioned drawbacks often lead to poor alignment of the fracture, resulting in more reduction process and treatment duration [13]. Furthermore, lower limb malalignment is an independent risk factor for knee osteoarthritis [14–17] and better alignment is conducive to the function reconstruction. With the development of imaging technology, three dimensional (3D) reconstruction technology has been widely used in medicine [18–20]. In fracture cases, the 3D reconstruction technology provides the axial information which cannot be presented on traditional X-rays.

In previous studies, markers installed on the rings were introduced to the process of 3D reconstruction [18–20]. In the present study, the adjustment plans can be automatically generated with the help of self-developed software, avoiding the potentially subjective errors using manual measurement. The purpose of this study was to propose a new marker-3D measurement method and determine the differences of reduction outcomes, if any, between marker-3D measurement method and traditional radiographic measurement in the TSF treatment, paving a way for many future works aiming to make the TSF process more efficient.

## Methods

### Study design and patients

Patients with tibial fracture treated by TSF in Tianjin Hospital were retrospectively analyzed from January 2016 to June 2019. The inclusion criteria were: (1) comminuted fracture (AO/Asif classification C3); (2) compound fractures (Gustilo type II / III); (3) the follow-up time after frame removal was ≥6 months. Exclusion Criteria were: (1) Patients with bilateral tibia fractures (unable to provide the mirror image of the contralateral three-dimensional reconstruction image); (2) patients unable to cooperate with regular follow-up. Finally, 41 patients were included in the study. There were 21 patients in the marker-3D measurement group (experimental group) and 20 patients in the traditional radiographic measurement group (control group).

### Measurement methods

All the treatment procedures were performed by the same surgical team. All patients underwent TSF installation as follows: the frame was fixed to the bone segment firstly with the struts in a sliding state, the fracture was preliminarily reduced by moving the rings under the C-arm followed by the lock of struts, residual deformities were corrected postoperatively by adjusting these struts.

#### Traditional radiographic measurement

**Fig. 1:**
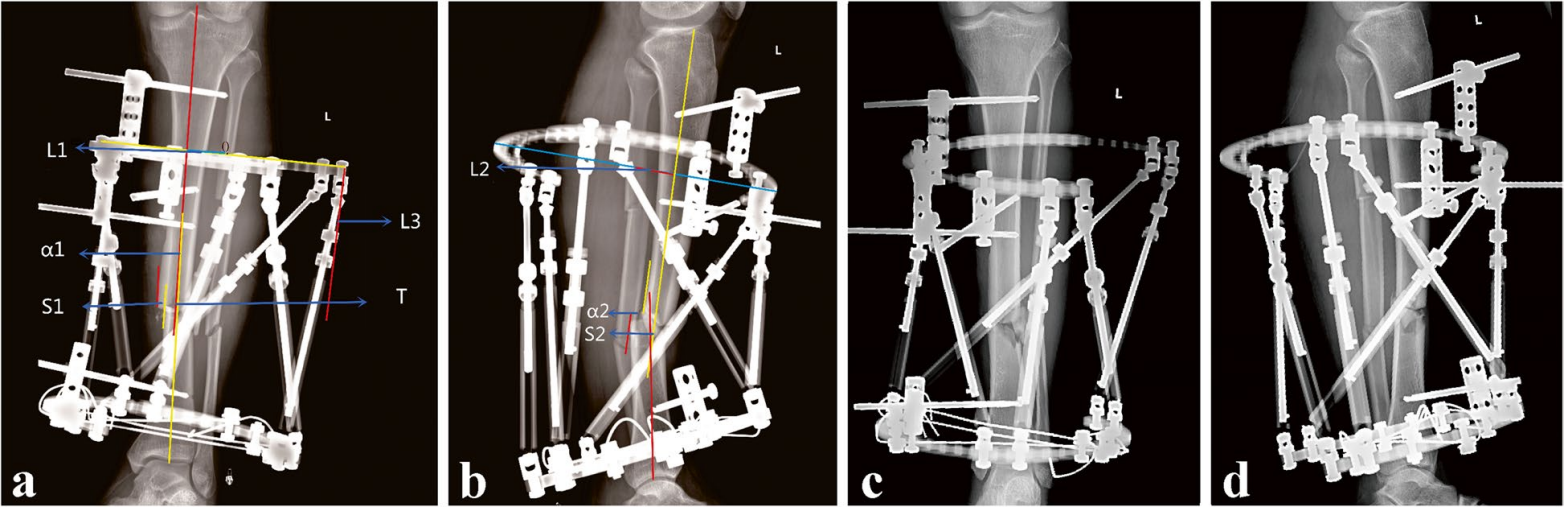
Radiographs showing patient with tibial fracture treated with TSF using traditional radiographic measurement method. Male,33 years old, left side. **a** Measuring deformity parameter in AP view. **b** Measuring deformity parameters in lateral view. **c** Immediate AP view after reduction. **d** Immediate lateral view after reduction

The postoperatively standard radiographs (AP and lateral radiographs, including proximal and distal joints as much as possible) of patients were conducted. The X-rays were imported into computer for parameters measurement (Fig. 1). The proximal bone segment was used as the reference, and the distal bone segment was determined as the free movement end. The midpoint of the proximal fracture line was taken as the center of rotation of angulation (CORA).

Parameters need to be measured include six deformity parameters and four mounting parameters according to the instructions. The deformity parameters include angulation and translation in the coronal (α1, S1), sagittal (α2, S2), and axial plane (physical examination, T). The mounting parameters which describe where the center of the reference ring is located relative to the origin point include anteroposterior view frame offset (L1), lateral view frame offset (L2), axial view frame offset (L3), and the rotary frame angle (physical examination) (Fig. 1).

#### Marker-3D measurement method

**Fig. 2:**
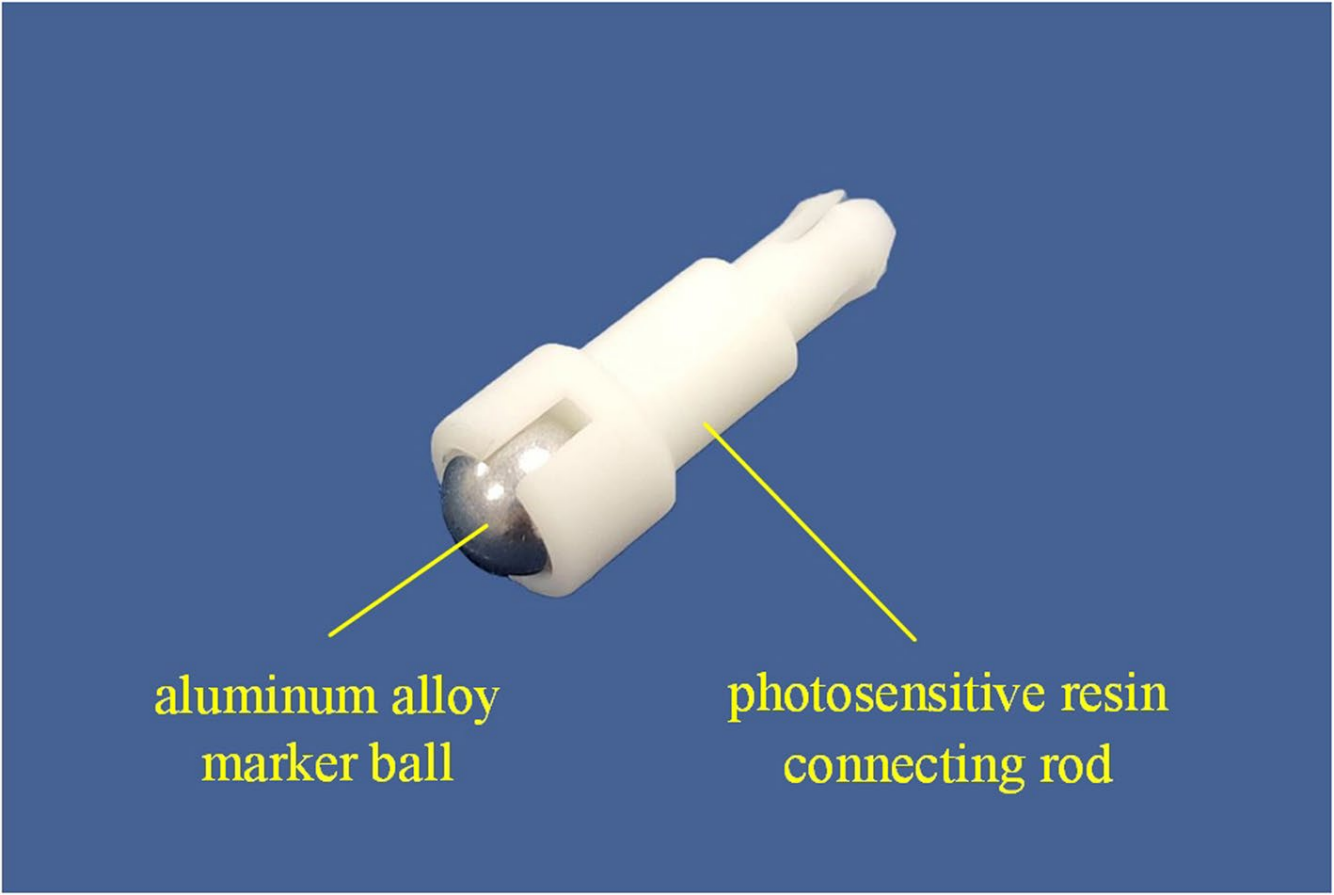
The composition of the marker

The marker was a composite structure, which was composed of aluminum alloy marker ball and photosensitive resin connecting rod (Fig. 2). A set of same markers were used for measurement.

##### 3D reconstruction

**Fig. 3:**
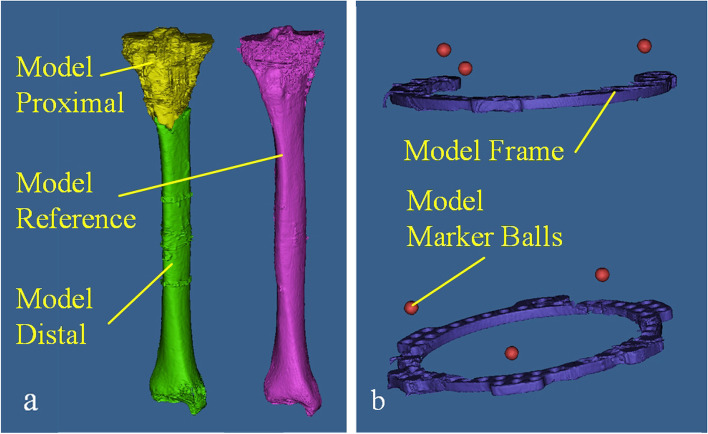
The 3D model of reconstruction. The fracture line was used as the boundary to divide the reconstructed affected limb bone model into the proximal and distal bone model. **a** Model Proximal (the 3D model of the proximal bone of the affected limb), Model Distal (the 3D model of the distal bone of the affected limb) and Model Reference (the 3D mirror model of healthy limb bone). **b** Model Frame (the 3D model of external fixation), and Model Marker Balls (the 3D model of Marker Balls)

Three markers were mounted on the proximal ring, the other three were mounted on the distal ring. The markers were distributed on each ring as evenly as possible (120 degrees). Bilateral lower limbs of each patient in the marker-3D measurement group underwent CT (GE Optima, CT66) scan for 3D reconstruction. The following models were generated: the 3D model of the proximal bony fragment of the affected limb (Model Proximal), the 3D model of the distal bony fragment of the affected limb (Model Distal), the 3D mirror model of healthy limb bone (Model Reference), the 3D model of external fixator (Model Frame), and the 3D model of Marker Balls (Model Marker Balls) (Fig. 3). The 3D mirror model of healthy limb was used for registration [21, 22].

##### Preparation in software

**Fig. 4:**
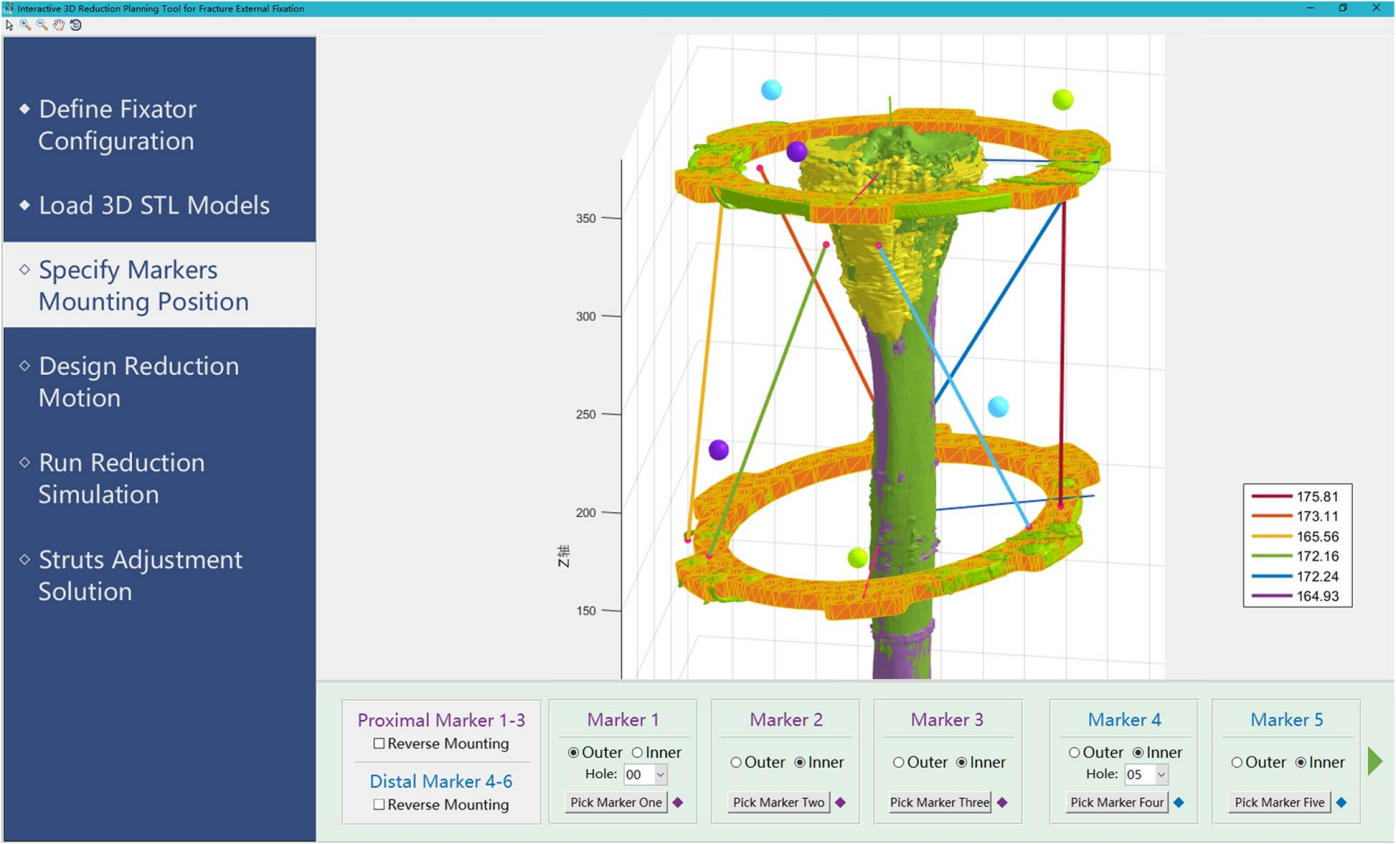
The interface of the 3D reduction software

The proximal/distal bony fragment and its relative ring was considered a rigid part respectively. A self-designed 3D reduction software was used for the measurement of electronic prescription (Fig. 4). The detailed marker locations on the ring needed to be inputted into the reduction software in which the spatial position of the marker balls can be recognized automatically as well as the initial position of the two rings. During the virtual fracture reduction, the software could automatically record the change in position.

##### Virtual fracture reduction using the custom software

**Fig. 5:**
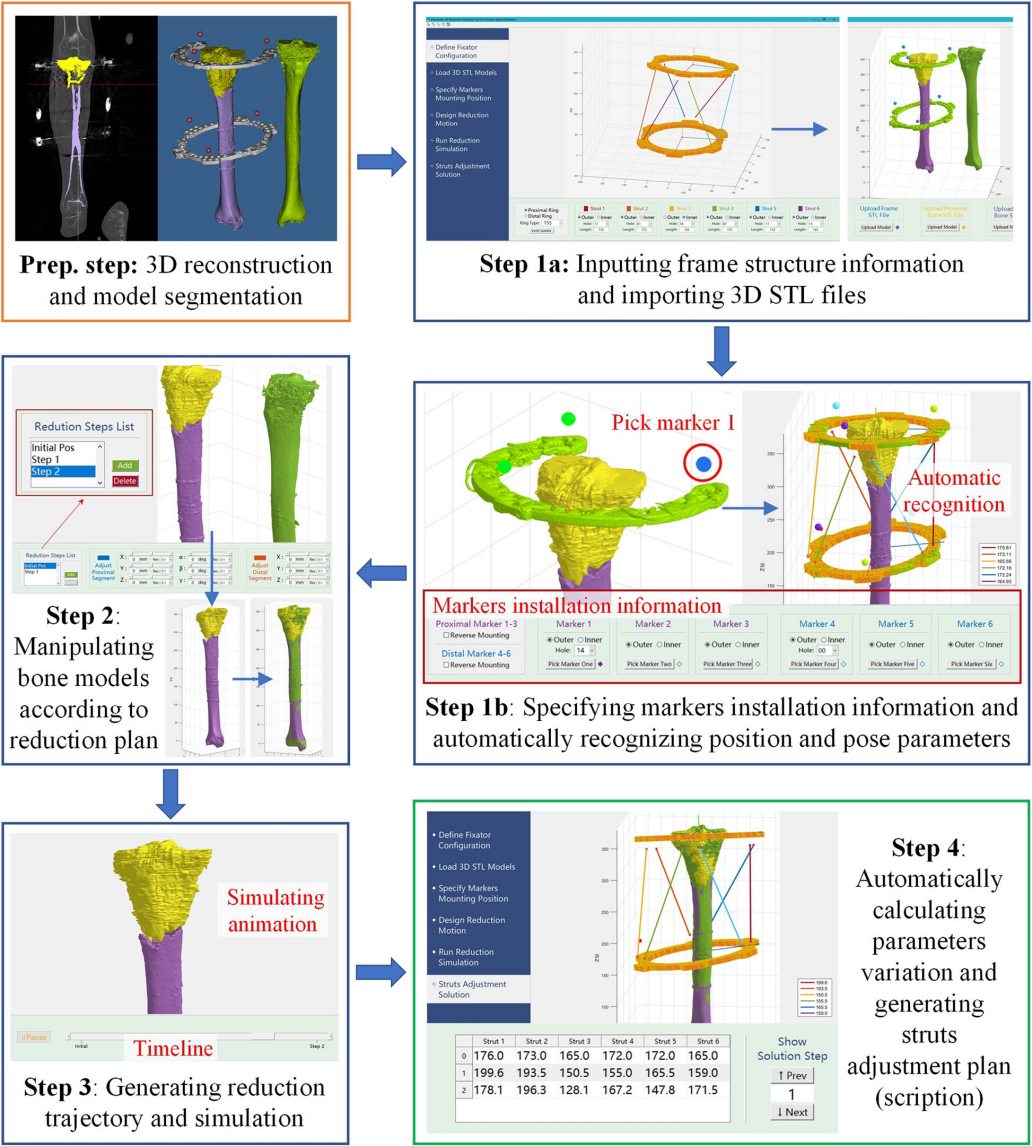
Schematic diagram of the marker-3D reconstruction method

**Fig. 6:**
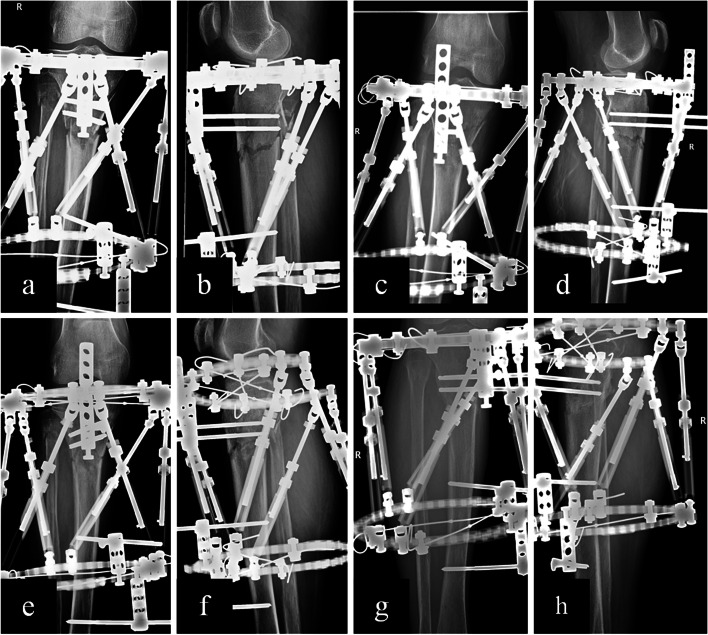
Radiographs showing patient with tibial fracture treated with TSF using marker-3D measurement method. Male,74 years old, right side. **a**, **b** Immediate AP and lateral view after surgery. **c**, **d** Immediate AP and lateral view after reduction. **e**, **f** One month later after surgery. **g**, **h** Three months later after surgery

(1) The reconstructed 3D models and the information of the frame and markers were imported into the custom software for virtual fracture reduction. (2) The protuberance tip on the bony segment and the feature point on the joint were used as the references. The Model Proximal was considered as the fixed end, the Model Distal was registered with the Model Reference to achieve fracture reduction directly. It was also possible to add multiple reduction intermediate points according to requirements. “traction-rotation-alignment” was the motion path of the bone to ensure the reduction safety. (3) The software could automatically generate the reduction path of the free movement end avoiding the collision between the bony segments. Furthermore, the virtual reduction animation was generated according to the initial and final position of the fixed ring. (4) The relative position changes of the two rings could be determined according to step 3. The length changes of the six struts were calculated by the Stewart mechanism kinematics algorithm, the strut ‘s adjustment plan (electronic prescription) was obtained finally. The schematic diagram was shown in Fig. 5. Typical case was shown in Fig. 6.

### Effectiveness evaluation

The effectiveness was evaluated by the residual displacement deformity (RDD) and residual angle deformity (RAD) in the coronal and sagittal plane, according to the standard AP and lateral X-rays after reduction.

### Statistical analysis

SPSS 22 (IBM Inc., New York, USA) was used for statistical analysis. The comparison between age was conducted by Student’s t test and represented as ^−^x ± s. The categorical data was compares by Chi-square test. The measurement data of abnormal distribution (residual deformities) was expressed as M (P25, P75) followed by Mann-Whitney U test. Significant difference was set as *P* ≤ 0.05.

## Results

### General information in two groups

All patients achieved functional fracture reduction and bone union. They were followed up at least 6 months after frame removal, and none was lost.

The experimental group comprised of 15 males and 6 females, with an average age of 49.5 ± 14.8 years (range 18 to 73 years). There were Gustilo classification Type II in 13 cases, Type III in 8 cases. Eleven patients with Type II wound were closed primarily within 8 h, while the other 2 Type II wound had delayed primary closure. Six patients with Type IIIA wound had split-thickness skin grafting, and the other 2 with Type IIIB wound were primarily managed by a monolateral fixator with a rotational flap and converted to TSF after 2 weeks.

The control group comprised of 17 males and 3 females, with an average of 47.6 ± 14.3 years (range19 to 76 years). There were Gustilo classification Type II in 14 cases and Type III in 6 cases. Thirteen patients with Type II wound were closed primarily within 8 h, while the other 1 Type II wound had delayed primary closure. Five patients with Type IIIA wound had split-thickness skin grafting, and the other 1 with Type IIIB wound were primarily managed by a monolateral fixator with a rotational flap and converted to TSF after 2 weeks.

The BMI of the experimental group and control group was 23.7 ± 2.4 kg/m^2^ and 23.4 ± 2.2 kg/m^2^ respectively. No statistical differences between the two groups in terms of gender, age, and fracture type were observed (*P* > 0.05) (Table 1).

**Table 1:** General information of two groups of patients

	Cases^a^	Gender^a^		Age^b^ (Years)	BMI^b^ (kg/m^2^)	Gustilo classification^a^	
		male	female			type II	type III
Experimental group	21	15	6	49.5±14.8	23.7±2.4	13	8
Control group	20	17	3	47.6±14.3	23.4±2.2	14	6
P^*^	-	0.50		0.67	0.73	0.59	

^a^Data are presented as number of patients

^b^Data are presented as mean ± standard deviation

*P values were calculated using Student’s t test and the Pearson chi-square test

### Residual displacement deformity (RDD)

The RDD of the experimental group in AP view was 0.5 (0, 1.72) mm, while the RDD of the control group in AP view was 1.74 (0.43, 3.67) mm. There was significant difference between the two groups (*P* = 0.024).

The RDD of the experimental group in lateral view was 0 (0, 1.22) mm, while the RDD of the control group in lateral view was 2.02 (0, 3.74) mm. Statistical significance was also observed between the two groups (*P* = 0.016) (Table 2).

**Table 2:** Comparison of residual deformities between the two groups after adjusting

Variable	Experimental group	Control group	*P* value*
M1^a^ (mm)	0.50(0,1.72)	1.74 (0.43, 3.67)	0.024
β1^b^ (°)	0(0,1.25)	1.25(0.62,1.95)	0.020
M2^a^ (mm)	0 (0, 1.22)	2.02 (0, 3.74)	0.016
β2^b^ (°)	0 (0, 0)	1.42 (0, 1.93)	0.004

M1 was the distance of the inward/outward movement in AP X-ray of the distal bone fragment relative to the proximal bone fragment; M2 was the forward/backward movement in lateral X-ray between the two bone fragments; β1 was the angle of varus/valgus in AP X-ray of the distal bone segment relative to the mechanical axis of the proximal bone segment; β2 was the angle of extension/flexion in lateral X-ray between the two bone fragments.

^a^Data and ^b^Data are presented as Median (P25, P75)

*P values were calculated using Mann-Whitney U test

### Residual angular deformity (RAD)

The RAD of the experimental group in AP view was 0(0, 1.25) °, and the RAD of the control group in AP view was 1.25 (0.62, 1.95) °. The difference between the two groups was statistically significant (*P* = 0.020).

The RAD of the experimental group in lateral view was 0 (0, 0) °, while the RAD of the control group in lateral view was 1.42 (0, 1.93) °. Statistical significance was also observed between the two groups (*P* = 0.004) (Table 2).

The residual deformities of displacement and angle in the experimental group were smaller than those in the control group, demonstrating the 3D-marker measurement method contribute to the satisfactory fracture reduction.

## Discussion

The present study proposed a method that can identify the spatial configuration of the frame automatically, providing advantages of reducing the measurement error and improving the reduction accuracy. According to Table 2, the residual deformities were significantly smaller in experimental group than that in control group, indicating the marker-3D measurement method could further improve the reduction accuracy compared to the traditional X-ray measurement method. The satisfactory results could come from the reduced measurement error and the definition of axial rotational deformity. Traditional method requires multiple measurements and adjustments to achieve satisfactory reduction [13], while in 3D measurement method, the satisfactory reductions could be achieved at the initial adjustment.

Abundant efforts have been developed to improve the TSF treatment. Simpson et al. used CT images for 3D reconstruction to perform virtual surgery [23]. He introduced a tracking stylus to digitize the connection holes as reference points on the TSF ring, or used the information of the bone surface for registration. This method avoided the measurement errors, but the result was greatly interfered by the choice of connecting holes. The positions of these connecting holes may be affected by the Kirschner wires and struts, resulting in the failure to find a suitable connection hole as the reference point. Furthermore, the imaging would be affected by metal artifacts.

Tang et al. designed a hexapod automatic fracture reduction device, similar to the Stewart platform, and then tested in animal models [21]. With the help of 3D reconstruction, 12 marker balls were used to replace 12 screw bolts, and the hinger’s length was directly identified by software followed by the automatical fracture reduction. However, this automatic reduction may ignore the soft tissue, blood supply, and the fracture shape during the reduction process, resulting in the collision of bony segments. Du H et al. redesigned the above device as a combination of a positioning unit, a reduction unit, and a control center [24]. Four non-special marking points of the positioning unit were used for registration to obtain the struts length. They introduced a series-parallel configuration to convert the 6-DOF movements of the hexapod mechanism into relevant movements of two holders, preventing the device from jamming during reduction and improving the portability of the device. However, there were several disadvantages in this design. Firstly, the positioning unit was composed of four parts which were complicated to disassemble and could lose precision during the process. Secondly, the various customized rings and devices may limit the clinical application of the device.

The standard TSF, an accessible marker, and a simply operated software were used in this study. The markers could be installed freely, and the software was able to automatically identify the position of the marker balls. With the help of the markers, the processes of measurement could be finished automatically, avoiding potential manual measurement errors. In addition, the electronic prescription for fracture reduction could be obtained through the final relative displacement transformation matrix using the operation-friendly software. With the help of CT data, the marker-3D measurement method could accurately obtain the axial information of the injured limb and generate the electronic prescription. In order to ensure the safety of the reduction, the self-developed software was able to examine the path of the reduction process. Two basic principles were used during fracture reduction: (1) bon traction and rotation need to be applied to avoid the collision of the fracture segments; (2) the bone segments are aligned with minimal movement to avoid overstretching of soft tissue. Previous studies have proved that the lower limb malalignment will increase the risk of knee osteoarthritis and medial meniscus lesions [14–17]. The marker-3D measurement method can effectively improve the alignment compared to the traditional X-ray method, providing the better clinical prognosis.

The use of markers to achieve automatic measurement also had the following shortcomings: (1) During CT scanning, the 3D reconstruction of the bone could be affected by the metal artifacts, affecting the reduction accuracy. (2) A conservative attitude should be adopted regarding the interpretations of our results due to a single-center small sample size. (3) The procedures are tedious and time-consuming in inexperienced hands. (4) Considering the higher radiologic exposure in CT than X-rays, our method is suggested to apply in those unusually complex cases. In the next step, the imaging technology needs to be improved to minimize the metal artifacts in the reconstruction process. The registration remained as a manual point-to-point registration in this study, automatic registration will be performed in the subsequent study. In addition, optical trackers and markers with automatic reduction robot system is our future direction.

## Conclusion

The present study introduced a marker-3D measurement method to complement the current TSF treatment. The marker is simple to application and compatible with the current mainstream external fixation instruments. This method avoids the manual measurement error and improves the accuracy of fracture reduction, providing potential advantages of bone healing and function rehabilitation.

## Data Availability

The datasets analysed during the current study are available from the corresponding author on reasonable request.

## Abbreviations

TSF: Taylor spatial frame
CT: Computed tomography
RDD: Residual displacement deformity
RAD: Residual angle deformity
AP: Anteroposterior
3D: Three-dimensional
CORA: Center of rotation of angulation
6-DOF: 6 degrees of freedom
OA: Osteoarthritis
